# Efficient synthesis of CRISPR-Cas13a-antimicrobial capsids against MRSA facilitated by silent mutation incorporation

**DOI:** 10.1038/s41598-024-67193-5

**Published:** 2024-07-13

**Authors:** Yuzuki Shimamori, Xin-Ee Tan, Feng-Yu Li, Yutaro Nishikawa, Shinya Watanabe, Teppei Sasahara, Kazuhiko Miyanaga, Yoshifumi Aiba, Srivani Veeranarayanan, Kanate Thitiananpakorn, Huong Minh Nguyen, Anujin Batbold, Tergel Nayanjin, Adeline Yeo Syin Lian, Sarah Hossain, Tomofumi Kawaguchi, Ola Alessa, Geofrey Kumwenda, Jayathilake Sarangi, Jastin Edrian C. Revilleza, Priyanka Baranwal, Mahmoud Arbaah, Maniruzzaman, Liu Yi, Ho Thi My Duyen, Takashi Sugano, Sharmin Sultana, Mohammad Omar Faruk, Yuya Hidaka, Myat Thu, Takayuki Shimojyo, Kotaro Kiga, Longzhu Cui

**Affiliations:** 1 Division of Bacteriology, Department of Infection and Immunity, School of Medicine, Jichi Medical University, Shimotsuke City, Tochigi 329-0498 Japan; 2 EIKEN CHEMICAL CO., LTD., Nogi, Shimotsuga District, Tochigi 329-0114 Japan; 3 Research Center for Drug and Vaccine Development, National Institute of Infectious Diseases, Tokyo, 162-8640 Japan

**Keywords:** Bacteriophages, Antimicrobial resistance, Biomedical engineering

## Abstract

In response to the escalating global threat of antimicrobial resistance, our laboratory has established a phagemid packaging system for the generation of CRISPR-Cas13a-antimicrobial capsids targeting methicillin-resistant *Staphylococcus aureus* (MRSA). However, a significant challenge arose during the packaging process: the unintentional production of wild-type phages alongside the antimicrobial capsids. To address this issue, the phagemid packaging system was optimized by strategically incorporated silent mutations. This approach effectively minimized contamination risks without compromising packaging efficiency. The study identified the indispensable role of phage packaging genes, particularly *terL-terS*, in efficient phagemid packaging. Additionally, the elimination of homologous sequences between the phagemid and wild-type phage genome was crucial in preventing wild-type phage contamination. The optimized phagemid-LSAB(mosaic) demonstrated sequence-specific killing, efficiently eliminating MRSA strains carrying target antibiotic-resistant genes. While acknowledging the need for further exploration across bacterial species and in vivo validation, this refined phagemid packaging system offers a valuable advancement in the development of CRISPR-Cas13a-based antimicrobials, shedding light on potential solutions in the ongoing battle against bacterial infections.

## Introduction

Clustered regularly interspaced short palindromic repeats (CRISPR), an acquired defense system of prokaryotes shielding them from unfavorable viral infections^1^, has revolutionized genetic engineering and molecular diagnostics, offering a versatile toolkit for applications such as gene editing (REPAIR)^2^, nucleic acid detection (SHERLOCK)^3^, and multiplexed RNA detection (LEOPARD)^4^. Within this dynamic landscape, the integration of CRISPR-Cas13a into antimicrobial strategies stands out as a promising approach to tackle antibiotic-resistant bacterial infections^5^. The clinical applicability of this CRISPR technology is attributed largely to the intrinsic collateral cleavage activity of certain subtypes of the CRISPR system, including, but not limited to, CRISPR-Cas13. When CRISPR-Cas13, primed with short CRISPR RNA (crRNA), recognizes and binds to its target RNA, a preceding specific RNA cleavage triggers conformational changes in the CRISPR-Cas13 protein to a non-specific RNase, resulting in indiscriminate cleavage of host RNAs, namely collateral cleavage^6^. This unique feature of CRISPR-Cas13 has been adopted by our laboratory for the development of an alternative antimicrobial agent capable of accomplishing sequence-specific killing of target antibiotic-resistant bacteria^5,7^.

Antibiotic-resistant infections pose serious public health threats, with the overuse of antibiotics which have been relied upon for decades, considered a major cause of the spread of antimicrobial resistance. The rapid emergence of resistant bacteria underscores the urgent need for new antibiotics. However, new classes of antibiotics have been lacking since the discovery of daptomycin and linezolid in the 1980s^8^. A recent antibiotic pipeline analysis released by the World Health Organization (WHO) highlights a concerning trend, revealing that over 80% of antibiotic candidates newly approved in 2020 are derivatives of existing antibiotic classes^9^. Recognizing the limitations of this trend, alternative therapeutic agents become of equal importance to effectively counter the rise of antimicrobial resistance. Currently, the development of a total of 27 non-traditional antibacterials is underway, encompassing nine antibodies, four bacteriophages and phage-derived enzymes, eight microbiome-modulating agents, two immunomodulating agents, and four miscellaneous agents^9^.

Our laboratory has previously engineered a phage-based non-traditional antibacterial agent, known as antibacterial capsids (AB-capsids), targeting methicillin-resistant *Staphylococcus aureus* (MRSA)^5^. These AB-capsids are non-replicative phage particles loaded with *mecA*-targeting CRISPR-Cas13a through our established phagemid packaging system (manuscript in preparation). The programmed CRISPR-Cas13a carried by phages is delivered into specific host bacteria, determined by the host range of the phage used in packaging, during normal viral transduction. Subsequent to this process, specific killing of MRSA is exhibited through collateral cleavage activity once the programmed CRISPR-Cas13a recognizes its target gene *mecA*^5^. The generation of AB-capsids is made efficient and straightforward with the utilization of the phagemid packaging system. However, certain challenges encountered during the construction of AB-capsids, such as contamination of wild-type phages and reduced AB-capsids titer, require attention. Low titers have the potential to impede the clinical applicability of AB-capsids, making it difficult to achieve a therapeutically effective dose. Additionally, the presence of wild-type phages poses potential issues, including: (1) loss of sequence specificity, as wild-type phages may kill host bacteria even if they do not carry the target sequence, and (2) the risk of undesirable horizontal gene transfer (virulence genes, toxin genes, etc.) through transduction. Addressing these challenges is crucial to ensuring the efficacy and safety of AB-capsids as a potential antibacterial therapeutic.

This study addresses these challenges by refining the phagemid packaging system, a pivotal step in the synthesis of AB-capsids. A key modification involves the strategic introduction of silent mutations within homologous sequences to effectively mitigate the contamination risk of wild-type phages without compromising packaging efficiency. This innovative approach not only resolves the challenges faced during the construction of AB-capsids but also enhances their potential as a formidable tool in the fight against antibiotic-resistant bacteria. The broader implications of this research go beyond the specific targeting of MRSA, offering comprehensive insights into the development and practical application of CRISPR-Cas13a-antimicrobial capsids.

## Results

### Evaluation of packaging genes essential for phagemid packaging and AB-capsid generation

**Figure 1:**
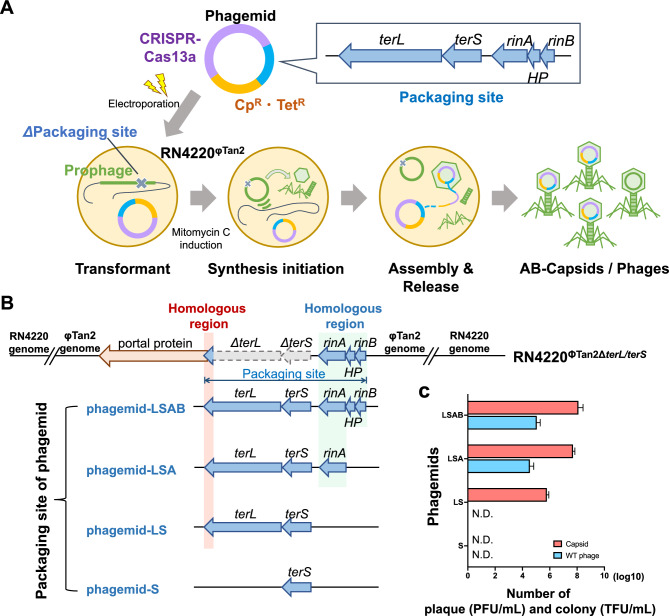
Influence of packaging genes on phagemid packaging and AB-capsid generation. (**A**) Schematic representation of phagemid packaging and AB-capsid generation. *S. aureus* cells with an integrated prophage Tan2 in their chromosome are transformed with the phagemid. The phagemid carries the CRISPR-Cas13a system, chloramphenicol resistance gene, tetracycline resistance gene, *ori* of *S. aureus* and *E. coli*, and the packaging gene(s). After transformation, mitomycin C induction activates phage synthetic processes, initiating the translation of phage structural proteins and leading to phage assembly. The phagemid is loaded into capsids, as it carries packaging signals that can be recognized during phage packaging machinery, thereby yielding AB-capsids. (**B**) Illustration depicting the packaging genes carried on the prophage genome of host *S. aureus* strain RN4220^ΦTan2Δ*terL*/*terS*^ and the phagemids. The upper panel shows the genome of the prophage integrated in host strain RN4220^ΦTan2Δ*terL*/*terS*^, with gray arrows indicating the knocked-out genes of *terL* and *terS*. The blue arrows represent the packaging gene(s) of the phagemids used in this experiment. The phagemid-LSAB, phagemid-LSA, phagemid-LS and phagemid-S corresponded respectively to phagemid pLK19::TerL-TerS-RinA-RinB, pLK19::TerL-TerS-RinA, pLK19::TerL-TerS, and pLK19::TerS, in our previous study (manuscript in preparation). The light red and light blue areas indicate homologous regions between prophage and phagemids. (**C**) Quantifications of transduced colony-forming unit (TFU) of AB-capsids and plaque forming unit (PFU) of wild-type phage packaged using host cell RN4220^ΦTan2Δ*terL*/*terS*^ transformed with any of the four phagemids (n = 3). Red bars indicate log TFU/ml, and blue bars represent log PFU/ml. Error bars represent standard deviations. *N.D.* not detected.

The phage-specific packaging site (pac), encoding signals for packaging phagemid into phage capsids to generate AB-capsids, comprises four genes: *terL*, *terS*, *rinA*, and *rinB*. We assessed the impact of knocking out pac genes from lysogenized Tan2 prophage on the efficiencies of phagemid packaging into phage capsids (Fig. 1A). In our previous study, the generation of AB-capsids using phage-lysogenized *S. aureus* strain with *terL* and *terS* deletion (RN4220^ΦTan2Δ*terL*/*terS*^) resulted in a relatively higher yield and lower levels of contamination with wild-type phages (manuscript in preparation). Here, we cloned different combinations of pac genes (*terS, terL-terS, terL-terS-rinA, or terL-terS-rinA-rinB*) onto phagemids and evaluated their efficacy in AB-capsid generation and preventing wild-type phage contamination when using RN4220^ΦTan2Δ*terL*/*terS*^ as the host cell (Fig. 1B). In brief, each phagemid was transformed into RN4220^ΦTan2Δ*terL*/*terS*^, and the transformants were chemically-induced with mitomycin C (MMC) to activate prophages. This in turn initiated phage synthetic processes resulting in the generation of AB-capsids (packaging of phagemids) and/or wild-type phages (packaging of phage DNA). As shown by the red bars in Fig. 1C, the three phagemids carrying the gene combinations of *terL-terS*, *terL-terS-rinA*, and *terL-terS-rinA-rinB* were packaged, generating AB-capsids with titers of 6.33 ± 1.89 × 10^5^, 5.17 ± 1.19 × 10^7^, and 1.53 ± 0.99 × 10^8^ TFU/ml, respectively. However, wild-type phages were also produced in the case of phagemids carrying *terL-terS-rinA* and *terL-terS-rinA-rinB* (blue bars), at approximately 4.00 ± 2.16 × 10^5^ and 1.27 ± 0.66 × 10^6^ PFU/ml, respectively. Notably, in the case of the phagemid carrying only *terL-terS*, no contamination with wild-type phages was observed; however, there was a significant reduction in AB-capsids yield. Furthermore, phagemids carrying only the *terS* packaging gene showed neither AB-capsids nor wild-type phage detection. This suggests that pac genes, especially *terL-terS*, are essential, while *rinA* and *rinB* are needed for efficient packaging of phagemid into the phage capsid.

### Removal of homologous sequences between prophage and phagemid

Upon learning that the entire pac genes are necessary for efficient packaging of the phagemid but lead to wild-type phage contamination, we hypothesized that the overlapping homologous region between prophage and phagemid might be a crucial contributing factor. To address this, we deleted the 57 bp long homologous sequence existing between the phagemid and Tan2 prophage in the host cell RN4220^ΦTan2Δ*terL*/*terS*^ (Fig. 1B), aiming to enhance the generation of AB-capsids while minimizing wild-type phage contaminations.

**Figure 2:**
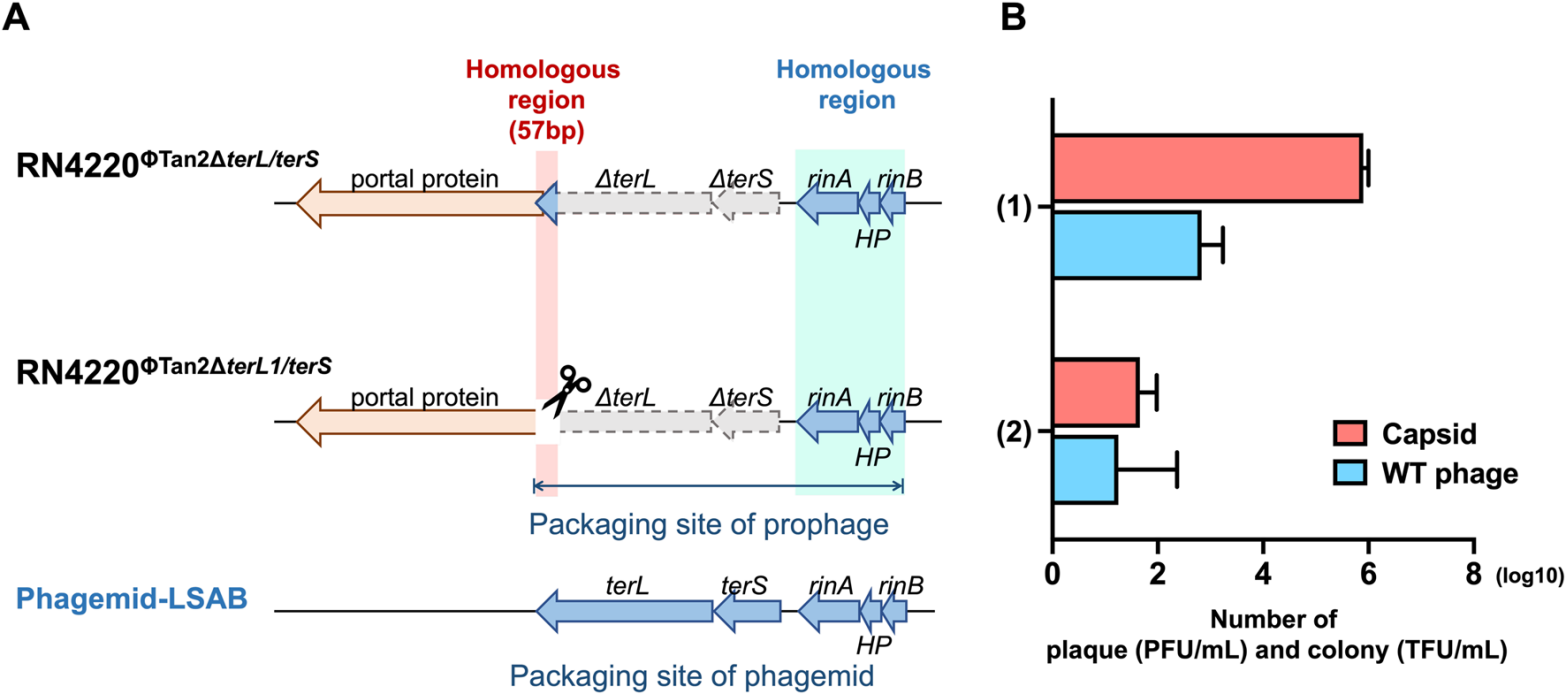
Deletion of homologous sequences existing between prophage and phagemid. (**A**) Schematic illustration of the packaging genes carried by phagemid-LSAB and the prophage genome on the host strain RN4220^ΦTan2Δ*terL*/*terS*^. The upper panel displays the host cell RN4220^ΦTan2Δ*terL*/*terS*^ used in our previous study (manuscript in preparation), while the lower panel shows the host cell RN4220^ΦTan2Δ*terL1*/*terS*^ with the deletion of a 57 bp homologous region used in the current study. The blue arrows indicate the packaging genes of the phagemid-LSAB. (**B**) Quantification of transduced colony-forming units (TFU) of AB-capsids and plaque-forming units (PFU) of wild-type phage packaged using both RN4220^ΦTan2Δ*terL*/*terS*^ and RN4220^ΦTan2Δ*terL1*/*terS*^ host cells transformed with phagemid-LSAB (n = 3). Red bars represent log TFU/ml, and blue bars represent log PFU/ml. Error bars represent standard deviations. *N.D.* not detected.

Initially, we generated a deletion mutant, RN4220^ΦTan2Δ*terL1*/*terS*^, by removing the 57 bp homologous sequence at the 3ʹ end of the *terL* gene in the prophage of RN4220^ΦTan2Δ*terL*/*terS*^ (Fig. 2A). We then transformed the mutant host cell, along with the original host cell RN4220^ΦTan2Δ*terL*/*terS*^, with phagemid-LSAB to produce AB-capsid. Subsequently, the production of both AB-capsids and wild-type phages was compared. Unexpectedly, the results demonstrated a significant decrease in the production of both AB-capsids and wild-type phages, from 7.87 ± 1.64 × 10^5^ to 5.33 ± 2.49 × 10^1^ TFU/ml and 9.33 ± 7.54 × 10^2^ to 6.00 ± 5.89 × 10^1^ PFU/ml, respectively (Fig. 2B). This finding indicates that the deletion of the 57 bp homologous sequence at the 3ʹ end of the *terL* gene is not viable for ensuring the efficient packaging of the phagemid-LSAB.

### Modification of the homologous sequences on phagemids

**Figure 3:**
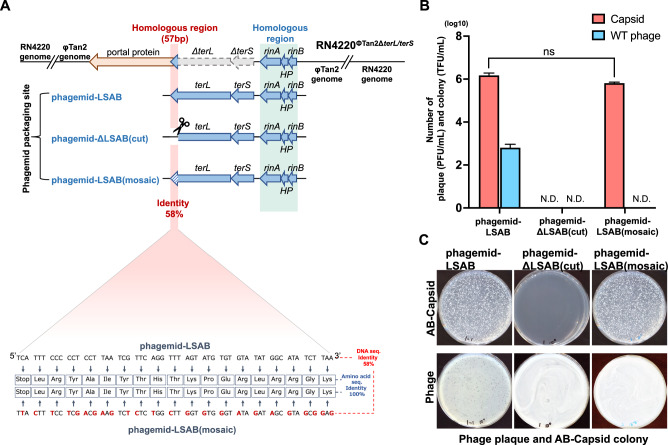
Homologous sequence modification and prevention of phage contamination. (**A**) Introduction of 24 silent mutations specifically into the packaging site of phagemid-LSAB(mosaic) to eliminate sequence homology. The upper panels show the packaging genes present on the three phagemids and the prophage of host RN4220^ΦTan2Δ*terL*/*terS*^. The lower panel displays the sequence alignment of the 57 bp homologous regions of phagemid-LSAB and phagemid-LSAB(mosaic), where the silent mutations were introduced. The light red area indicates the 57 bp homologous regions. (**B**) Quantification of transduced colony-forming units (TFU) of AB-capsids and plaque-forming units (PFU) of wild-type phage packaged using host cell RN4220^Φtan2Δ*terL*/*terS*^ transformed with different phagemids (n = 3). Error bars represent standard deviations. N.D.; not detected. Student’s *t*-test was performed to determine the significant difference between AB-capsids titer produced using phagemid-LSAB or phagemid-LSAB(mosaic). ns, not significant. (**C**) Representative agar plates with bacterial colonies formed by AB-capsids transduction (upper) and plaques formed by contaminated wild-type phages (lower).

Deletion of the 57 bp homologous region shared between phagemid-LSAB and the prophage in RN4220^ΦTan2Δ*terL*/*terS*^ is not plausible considering that this 57 bp sequence is situated within the overlap region of the end of *terL* and the beginning of a neighboring gene that encodes a portal protein. The deletion of this 57 bp region resulted in the removal of the initial codon of the portal gene, rendering it dysfunctional. As an alternative way to eliminate sequence homology between prophage and phagemids, we introduced silent mutations into the 57 bp homologous region of *terL*, replacing 24 nucleotides without causing amino acid substitutions. This modification reduced nucleic acid sequence identity to about 58%, resulting in the creation of phagemid-LSAB(mosaic) (Fig. 3A, Table S4). Additionally, the phagemid-ΔLSAB(cut) with a deletion of the 57 bp sequence was prepared as a control, other than phagemid-LSAB. Using these three phagemids, we compared their efficacy in the production of AB-capsids and prevention of wild-type phage contamination. Surprisingly, as depicted in Fig. 3B and C, phagemid-LSAB(mosaic) yielded 6.53 ± 0.61 × 10^5^ TFU/ml of pure AB-capsids with undetectable levels of wild-type phage contamination. The AB-capsids titer showed no significant difference compared to that produced by phagemid-LSAB (1.51 ± 0.35 × 10^6^ TFU/ml). As anticipated, phagemid-ΔLSAB(cut) generated neither AB-capsids nor wild-type phage.

### Sequence-specific killing of phagemid-LSAB(mosaic)-based AB-capsids against *S. aureus*

Considering the above discoveries, our focus shifted towards exploring the sequence-specific killing capabilities of the generated AB-capsids against *S. aureus* by targeting various important/frequently-encountered antibiotic resistant genes to address clinical needs. The phagemid-LSAB(mosaic) harbors the chloramphenicol resistance (CpR) gene *cpr* from pIMAY, which functions in both *S. aureus* and *E. coli*^10^. Additionally, it carries the tetracycline resistance (TetR) gene *tetM* from SaPIbov2 bap::tet30^11^. The inclusion of double antibiotic resistance markers eases the screening of AB-capsids killing activities across a wide spectrum of clinical *S. aureus* strains, considering that the 500 clinical isolates examined rarely possess both genes simultaneously. In addition to the *mecA* gene, we selected the following eight antibiotic-resistant genes of *S. aureus* as preliminary target genes to test the sequence-specific bacterial killing effect of AB-capsids against antibiotic-resistant *S. aureu*s: *aph(2ʹ), aadD, aph(3ʹ), aac(6ʹ), ermB, fusC, mphC,* and *tetK* (Table S1). Importantly, *aph(2ʹ), aph(3ʹ)*, and *aac(6ʹ)* are associated with gentamicin and tobramycin resistance (aminoglycoside antibiotics ); *aadD* with streptomycin resistance (aminoglycoside antibiotics); *ermB* with erythromycin resistance (macrolide antibiotics); *fusC* with fusidic acid resistance; *mphC* with resistance towards macrolides; and *tetK* towards tetracyclines.

**Figure 4:**
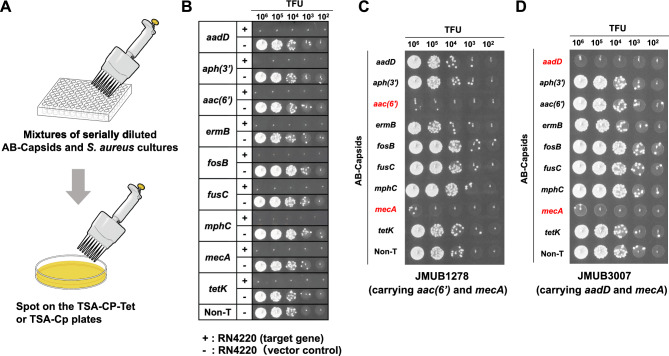
Sequence-specific bacterial killing by AB-capsids generated using modified phagemid-LSAB(mosaic). (**A**) Schematic representation of the experimental flow for evaluating the bacterial killing activity of AB-capsids generated using phagemid-LSAB(mosaic) in this study. A tenfold serial dilution of AB-capsids was mixed with bacterial cultures of strains carrying target genes, and then 2.5 µl of the mixtures were spotted on TSB agar plates containing chloramphenicol and tetracycline to visualize bactericidal activity. (**B**) Sequences-specific bacterial killing of the AB-capsids was evaluated using target gene overexpressed transformants of *S. aureus* RN4220. The genes overexpressed in RN4220 are listed in the left columns, and the presence or absence of the target gene in the bacterium are shown with ( +) and ( −), respectively. (**C**,**D**) Sequence-specific bacterial killing of the AB-capsids against clinical isolates of methicillin-resistant *S. aureus* (MRSA). Besides *mecA*, JMUB1278 carries *aac(6ʹ)* associated with gentamicin and tobramycin resistance (aminoglycoside antibiotics); and JMUB3007 carries *aadD* that is associated with resistance to streptomycin (aminoglycoside antibiotics). Antibiotic resistance genes present in clinical isolates are highlighted in red font. Each gene listed on the left plane represents the different resistant gene-targeting AB-capsids used, with Non-T representing the non-targeting AB-capsid that lack spacers as control.

In each instance, 25 bp long spacers complementing each of the aforementioned genes were designed and inserted into the phagemid to generate a resistance gene-targeting phagemid-LSAB(mosaic) series. The non-targeting phagemid-LSAB(mosaic) without a spacer (Non-T) serves as a negative control. Subsequently, each phagemid was transformed into RN4220^ΦTan2Δ*terL*/*terS*^ to produce AB-capsids targeting the specified antibiotic-resistant genes mentioned above. Sequence-specific bactericidal activity of the resultant AB-capsids were then evaluated using spot assay (Fig. 4A). Notably, all nine different AB-capsids exhibited pronounced bactericidal activity against their corresponding target *S. aureus* strains. These strains include both the transformants of RN4220, where each individual target gene was over-expressed on plasmid pKAK (Fig. 4B), and clinical isolates JMUB1278 and JMUB3007, which carry antibiotic-resistant genes *aac(6ʹ)* and *mecA* (Fig. 4C), and *aadD* and *mecA* (Fig. 4D), respectively. The specified antibiotic-resistant genes targeted by the generated AB-capsids are highlighted in red.

## Discussion

Antimicrobial resistance has emerged as a global health threat, partly due to the stagnation in antibiotic development. To tackle this issue, there has been a renewed interest in phage therapy, coupled with innovative strategies like phage-antibiotic combinations, phage-derived enzymes, and phage bioengineering^12,13^. The application of natural or engineered phages, either alone or in combination with traditional antibiotics, in compassionate therapies or clinical trials targeting infections with *Acinetobacter baumannii*^14^, *Mycobacterium abscessus*^15^, *S. aureus*^16^, and *Pseudomonas aeruginosa*^17^ further supports the feasibility of phage therapy in clinical settings. In our laboratory, we have developed AB-capsids, a novel non-traditional antibacterial agent designed to combat the multidrug-resistant MRSA strains^5^. These AB-capsids are non-replicative phage particles loaded with *mecA*-targeting CRISPR-Cas13a through our established phagemid packaging system, demonstrating promising in vitro results. However, the translation from controlled laboratory environment to the dynamic realm of clinical applications poses substantial challenges.

The shift from experimental setup to clinical application necessitates careful consideration of several critical factors before deploying natural or engineered phages. These factors include: (1) specificity and efficiency in eradicating pathogenic bacteria, (2) ease of preparation to achieve high titer and purity, (3) stable storage without substantial deterioration in bactericidal activity, and (4) safety concerns, ensuring the absence of endotoxins or harmful contaminants and preventing undesirable horizontal gene transfer^18^ While the phagemid packaging system offers a promising avenue for the efficient generation of AB-capsids, this current production system faces issues such as contamination with wild-type phages and low AB-capsid titers, necessitating refinement to enhance efficiency and reliability. Addressing these challenges is therefore essential to advance the clinical potential of AB-capsids.

In this study, AB-capsids were generated using RN4220^ΦTan2Δ*terL*/*terS*^, a phage-lysogenized *S. aureus* strain with *terL* and *terS* deletion, to minimize the risk of wild-type phage contamination. The TerS-TerL complexes, crucial components in phage DNA packaging^19^, were identified as pivotal in this process. It has been documented that the complete elimination of natural phage production can be achieved through the deletion of prophage *terS*^20^. However, despite these measures, wild-type phage contamination was observed during the packaging of phagemid-LSAB and phagemid-LSA using RN4220^ΦTan2Δ*terL*/*terS*^. This phenomenon was traced back to the presence of homologous regions, both at the 5ʹ end (*rinA* or *rinA-rinB*) and 3ʹ end (57 bp sequence) of phage-encoded packaging site genes, shared between phagemids and prophage (Figs. 1B, 2A). It is postulated that these homologous sequences compensate for the loss of *terS* and *terL* on the prophage, facilitating the concatemerization of natural phage DNA^21,22^ and subsequent packaging of natural phages. As evidenced in the packaging of phagemid-LS, the removal of *rinA*-*rinB* successfully abolished 5ʹ end homology, consequently eliminating wild-type phage contamination. However, the absence of *rinA* adversely affected AB-capsid production (Fig. 1C). Ferrer et al. previously reported that *rinA* plays a crucial role as a regulator of two major gene clusters of phages, specifically the morphogenesis and lysis clusters^23^. Deletion of *rinA* resulted in a significant reduction in phage/phage-like particles. Given these findings, the presence of *rinA* on phagemids is believed to be advantageous in our phagemid packaging system, contributing to the optimal production of AB-capsids.

As an alternative strategy to address wild-type phage contamination, a 57 bp homologous sequence on the 3ʹ end of *terL* gene in the prophage of RN4220^ΦTan2Δ*terL*/*terS*^ was removed. The deletion of the 3ʹ end homologous region on the prophage proved detrimental, resulting in a significant reduction in AB-capsid titers from 7.8 × 10^5^ TFU/ml to 5.33 × 10^2^ TFU/ml (Fig. 2B). Notably, the 3ʹ end homologous region encompasses the initial codon of a gene encoding the phage portal protein (Figs. 1B, 2A). The portal protein serves as the docking subunit for TerL, initiating phage DNA packaging^24^. The interaction between TerL and the portal protein is crucial for the assembly of phage capsids, particularly in tailed phages^25^. On the other hand, removal of 3ʹ end homologous region from phagemids is undesirable, considering the importance of C-terminal of *terL* in cleaving phage DNA during packaging^26–28^. Moreover, while controversial, the C-terminal of *terL* is proposed to be the domain that binds to portal protein to initiate phage DNA packaging due to its closer proximity to the portal compared to its N-terminal ends^29^. Complete elimination of the 3ʹ end homologous region, whether from the prophage or phagemids, is thus deemed inappropriate (Figs. 2, 3).

Subsequently, we employed a strategy involving the introduction of silent mutations into the 3ʹ end homologous region on phagemids. Forty-two silent mutations were intricately incorporated into the C-terminal of *terL*, replacing 24 nucleotides without inducing amino acid substitutions. This modification resulted in the creation of a phagemid termed phagemid-LSAB(mosaic) (Fig. 3A, Table S4), which facilitated the production of high titers of AB-capsids while completely eliminating wild-type phage contamination (Fig. 3B). Notably, this highlighted that a reduction in sequence similarity at the 57 bp region between prophage and phagemids to approximately 58% proved sufficient to impede recombination between the two DNA sequences and hinder natural phage production. These results indicated that we successfully optimized the phagemid packaging system, addressing the complex interaction between the packaging site on the phagemid and the prophage in host cells. The AB-capsids generated from RN4220^ΦTan2Δ*terL*/*terS*^ using phagemid-LSAB(mosaic) were subsequently subjected to testing against *S. aureus* strains carrying targeted antibiotic-resistant genes (Fig. 4A). The applicability of phagemid-LSAB(mosaic)-based AB-capsids was not merely confined to the laboratory-derived strains, instead their sequence-specific killing efficacy were also demonstrated using clinical isolates of *S. aureus* strains carrying different target antibiotic-resistant genes (Fig. 4B, C). While acknowledging the need for further exploration into crucial aspects such as stability and safety, this study presented a refined phagemid packaging system effective in generating phage-based antibacterials with improved titers and purity, marking a significant stride toward realizing the potential of phage therapy.

While this study marks a significant advancement in targeted antimicrobial agents development, it is crucial to acknowledge its inherent limitations. The specificity of the AB-capsids, primarily demonstrated against MRSA, raises questions about its generalizability to diverse bacterial species. The complexity of bacterial strains and their potential variations in response to the optimized system necessitate thorough examination across multiple strains. Additionally, the lack of in vivo validation poses a notable limitation. While in vitro experiments lay the groundwork for understanding the system’s performance, translating these findings into relevant animal models or clinical settings is essential. In vivo validation would provide insights into the practicality and effectiveness of the system in complex biological environments, and address concerns about potential off-target effects. Despite improvements in titers and purity, future research addressing stability and safety concerns associated with the phagemid packaging system is necessary.

In conclusion, the optimization of the phagemid packaging system through the incorporation of silent mutations represents a significant advancement in the development of CRISPR-Cas13a-antimicrobial capsids against MRSA. The study’s findings not only contribute valuable insights into combating antibiotic resistance but also pave the way for future research exploring the diverse applications and potential collaborations in the dynamic field of CRISPR-based antimicrobials. While the study provides profound insights, recognizing and addressing its limitations through continued research and refinement is paramount for unlocking the full translational potential of these findings.

## Methods

### Bacterial strains and culture conditions

*S. aureus* strains were cultivated at 37 °C in tryptic soy broth (TSB; BD Difco, USA) or on tryptic soy agar (TSA; BD Difco, USA). Chloramphenicol (Cp) at a concentration of 10 µg/ml, and/or Tetracycline (Tet) at 5 µg/ml was added when necessary. *E. coli* strains were grown at 37 °C in Luria–Bertani (LB) broth (BD Difco, USA) or on LB agar (BD Difco, USA), with the addition of following antibiotics when appropriate: 30 µg/ml of kanamycin (Km), 100 µg/ml of ampicillin (Amp), 5 µg/ml of Tet and/or 10 µg/ml of Cp. All *S. aureus* and *E. coli* strains used in this study were listed in Supplementary Table S1^10,30^.

### Construction of pac deletion mutants *S*. *aureus* RN4220^ΦTan2ΔterL/terS^ and RN4220^ΦTan2ΔterL1/terS^

0The RN4220^ΦTan2Δ*terL/terS*^ and the RN4220^ΦTan2Δ*terL1/terS*^ mutants were generated through allelic exchange using temperature-sensitive plasmids. The RN4220^ΦTan2Δ*terL/terS*^ mutant, with deletions of the t*erL* and *terS* genes from Tan2 prophage, was generated with the plasmid pIMAY^10^, as described in our previous study (manuscript in preparation). In this study, we aimed to generate another mutant derivative of RN4220^ΦTan2 WT^, RN4220^ΦTan2Δ*terL1/terS*^. This new mutant has a deletion of a 57 bp region at the 3ʹ end of the *terL* gene. We generated this mutant using the plasmid pKAK^31^ through allelic exchange. For the construction of RN4220^ΦTan2Δ*terL1*/*terS*^, 500–1000 bp upstream and downstream flanking sequences of *terL-terS* of prophage Tan2 were amplified with two different primer sets: pKFT KO SL no promoter S1 v2/pKFT KO SL no promoter AS1 v2 and pKFT KO SL no promoter S2 v2/pKFT KO SL single and no promoter AS2, employing KOD FX Neo polymerase (Toyobo, Japan). The pKFT^31^ plasmid was also PCR-amplified using the same polymerase with primer set pKFT MCS outside S/pKFT MCS outside AS. The three PCR-amplified fragments were subsequently ligated using NEBuilder^®^ HiFi DNA Assembly (New England Biolabs, USA) to ultimately generate pKFT_ΔTerL/TerS knockout plasmid, which was then transformed into *E. coli* DC10B and selected on LB agar supplemented with 100 µg/ml of Amp.

The constructed plasmid was extracted and verified by Sanger sequencing. Following that, sequences-verified knockout plasmids were electroporated into RN4220 integrated with prophage Tan2 using ELEPO21 electroporator (Nepa Gene, Japan). The following pulse parameters were used: Poring Pulse (voltage: 1800 V, pulse length: 2.5 ms, pulse interval: 50 ms, number of pulses: 1, Polarity: +); Transfer Pulse (voltage: 100 V, pulse length: 99 ms, pulse interval: 50 ms, number of pulses: 5, polarity: + / −)^32^. The transformants were grown on TSA supplemented with 5 µg/ml Tet at 30 ℃. Single crossover was achieved by growing the cells on TSA supplemented with 5 µg/ml Tet at non-permissive temperature of 43 ℃. Finally, double crossover was performed by growing the cells on TSA supplemented with 5 µg/ml anhydrotetracycline at 37 ℃. The construction of *S. aureus* RN4220^ΦTan2Δ*terL1*/*terS*^ mutants was further confirmed by PCR and Sanger sequencing.

All primer and oligo DNA sequence used in this study were listed in Table S2, all plasmids used in this study were listed in Table S3^31,33^.

### Construction of phagemid-ΔLSAB(cut) and phagemid-LSAB(mosaic)

The phagemid-ΔLSAB(cut) and phagemid-LSAB(mosaic) were constructed based on phagemid-LSAB (manuscript in preparation). Phagemid-LSAB is a *S. aureus*-*E. coli* shuttle vector cloned with CRISPR-Cas13a. It consists of CRISPR-Cas13a, antibiotic selection marker KmR (*aphA-3*) and *E. coli* ori amplified from pKLC21, *S. aureus* ori and antibiotic selection marker CpR (*cat*) amplified from pKLC14, and packaging sites *rinB-rinA-terS-terL* amplified from Tan2 phage. In our previous study, we have used the phagemid-LSAB, which includes the CRISPR-Cas13a system, CP resistance gene, Tet resistance gene, *ori* of *S. aureus* and *E. coli*, and the Tan2 packaging genes, for the generation of AB-capsids.

For the construction of phagemid-ΔLSAB(cut), phagemid-LSAB was linearized, with the exclusion of a 57 bp sequences at the 3ʹ end of *terL*, through PCR amplification using primers pLK19_1_F/pLK19_1_R. Subsequently, the amplified fragments were self-ligated using NEBuilder^®^ HiFi DNA Assembly to finally generate phagemid-ΔLSAB(cut). On the other hand, phagemid-LSAB(mosaic) was constructed by ligating two PCR-amplified fragments of phagemid-LSAB with a synthesized 57 bp mosaic sequence fragment, where 24 silent mutations (without amino acid substitution) were introduced, using NEBuilder^®^ HiFi DNA Assembly.

All constructed phagemids were subsequently transformed into *E. coli* DC10B and selected on LB agar supplemented with 10 µg/ml Cp. Successful transformation of each phagemid was validated by colony PCR. Following validation, the phagemids were extracted using FastGene^®^ plasmid mini kit (NIPPON Genetics, Japan).

### Generation of phagemid-based staphylococcal AB-capsid

The constructed phagemids [phagemid-ΔLSAB(cut) and phagemid-LSAB(mosaic)] were individually transformed into host cell *S. aureus* RN4220^ΦTan2Δ*terL*/*terS*^ by electroporation using ELEPO21 electroporator (as previously described)^32^. Resulting transformants were recovered at a temperature permissive for plasmid replication (30 °C) for 5 h and then plated on TSA plates supplemented with Cp. Successful transformation of each phagemid was validated by performing colony PCR. Next, single PCR-confirmed colonies were inoculated in TSB containing Cp and cultured at 37 °C with shaking. Mitomycin C (FUJIFILM Wako Pure Chemicals, Japan) was added to a final concentration of 0.4 µg/ml when the bacterial culture reached OD600 of 0.5 to initiate the phage synthesis process, and subsequently, the phagemid DNAs were packaged, and AB-capsids were produced. Mitomycin C induction was carried out for 7 h at 30 °C with shaking at 80 rpm. After incubation, the culture was centrifuged and the supernatant removed. The pellet was then suspended in 1 ml of TSB containing Cp and incubated overnight at 30 °C. On the following day, the culture was added with another 1 ml of TSB containing Cp and centrifuged to pellet cell debris. The supernatant containing phagemid-based staphylococcal AB-capsids were passed through a 0.22 µm membrane filter and the AB-capsids solution was harvested.

### Measurement of titers of phage and AB-capsid

The AB-capsid solution was serially tenfold diluted with SM buffer to a range of 10^−1^ to 10^−7^. Meanwhile, an overnight culture of *S. aureus* strain RN4220, diluted 1:100 with TSB broth, was incubated with agitation at 37 °C until an OD_600_ of approximately 0.5. Then, 110 µl of each dilution of AB-capsid solution was added to an equal volume of bacterial suspension, and the mixture was incubated at 37 °C for 20 min. After that, 100 µl of the mixture was added to 4 ml of soft top agar (TSB solution with 0.75% agarose) pre-warmed at 55 °C and overlaid on a TSA plate, and a Cp TSA plate, respectively. The solidified plates were incubated at 37 °C overnight. The colonies grown on the Cp TSA plate were counted to calculate the transduced colony-forming units, TFU/ml; whereas the plaques formed on the drug-free TSA plate were counted to calculate the plaque-forming units, PFU/ml. All assays were repeated three times (n = 3).

### Spacers insertion for targeted bacterial killing

The CRISPR-Cas13a system cloned on phagemid-LSAB(mosaic) was designed to target nine drug-resistant genes: *aadD, aph(3ʹ), aac(6ʹ), ermB, fosB, fusC, mphC, mecA,* and *tetK*. The 25 bp long spacer sequences targeting each of the above genes, and a non-targeting spacer (irrelevant sequence) included as a control, were selected. A total of 10 types of 85-mer oligo DNAs, comprising a 25-mer spacer sequence (crRNA) complementary to the conserved region of each target gene, flanked upstream and downstream each with 30-mer nucleotides corresponding to the consensus sequences at the point of insertion on the phagemid, were custom-designed. After that, phagemid-LSAB(mosaic) was treated with the restriction enzyme *Bsa*I-HF, purified with gel electrophoresis, and subsequently ligated with the 85-mer oligo DNAs using NEBuilder^®^ HiFi DNA Assembly to obtain phagemid-LSAB(mosaic)-X (X represents the target gene). Following the assembly of the phagemids, AB-Capsid_X were generated through mitomycin C induction as aforementioned.

### Construction of target gene overexpression plasmids

In our previous study, we constructed a *mecA* gene-overexpression plasmid using the pKAT plasmid^33^. Briefly, the *mecA* gene was amplified by PCR with the J1417/J1418 primer set from the genomic DNA of the clinical MRSA isolate JMUB217^34^. Subsequently, pKAT::*mecA* was constructed by treating the PCR fragment and the pKAT plasmid with *Sal*I and *Sma*I restriction enzymes, followed by ligation of the two resulting DNA fragments. For the purpose of our current study, Cp resistance gene of pKAT::*mecA* was replaced with Km resistance gene to generate pKAK::*mecA*. Briefly, the Km resistance gene was PCR-amplified with the aacF/aac-R primer set using the genomic DNA of JMUB217 as template, while pKAT fragment without Cp resistance gene was amplified by PCR using the aac-F-pKAT-F/aac-F-pKAT-R primer set. The amplified fragments were assembled using NEBuilder^®^ HiFi DNA Assembly, resulting in the generation of pKAK::*mecA*.

Other target gene overexpression plasmids were then constructed in a similar way using pKAK plasmid backbone. To prepare the pKAK fragment, *mecA* gene was excluded from pKAK::*mecA* plasmid by PCR amplification using the pKAK_YS_F/pKAK_YS_R primer set. Simultaneously, target gene fragments were PCR-amplified from the genomic DNA of corresponding clinical *S. aureus* strains of which whole genome sequences have been determined. The primer sets used for amplifying each gene are listed in Supplementary Table 1. Each amplified fragment was assembled with the plasmid backbone using NEBuilder^®^ HiFi DNA Assembly, resulting in the generation of different target gene overexpression plasmids.

### Construction of target gene overexpression strains

The constructed target gene overexpression plasmids were individually transformed into *S. aureus* RN4220 by electroporation with ELEPO21 electroporator using the same pulse parameters as described previously^32^. The resulting transformants were recovered at a temperature permissive for plasmid replication (30 °C) for 5 h and then plated on TSA plates supplemented with Km. Successful transformation of each expression plasmids were validated by colony PCR.

### Bacterial killing assay

The ability of the generated AB-capsids to sequence-specifically kill target bacteria was determined using a spot test assay. The *S. aureus* strains to be assayed, including selected in-house whole-genome-sequenced *S. aureus* clinical isolates and transformants of *S. aureus* RN4220 overexpressing target gene, were grown to an OD_600_ of approximately 0.5. In the meantime, AB-capsids were tenfold serially diluted. Each dilution of the AB-capsids was then mixed with the studied strains in a 1:1 ratio and incubated for 20 min. Finally, 2.5 µl of each mixture was spotted onto CP TSA or CP Tet TSA plates, and the plates were incubated at 37 °C overnight. The assay was repeated three times (n = 3).

### Supplementary Information

Supplementary Information.

## Data Availability

The datasets used and/or analyzed during the current study are available from the corresponding author upon reasonable requests.
